# Antimicrobial Coatings Based on Hybrid Iron Oxide Nanoparticles

**DOI:** 10.3390/nano15090637

**Published:** 2025-04-22

**Authors:** Doina-Antonia Mercan, Dana-Ionela Tudorache (Trifa), Adelina-Gabriela Niculescu, Laurenţiu Mogoantă, George Dan Mogoşanu, Alexandra Cătălina Bîrcă, Bogdan Ștefan Vasile, Ariana Hudiță, Ionela Cristina Voinea, Miruna S. Stan, Tony Hadibarata, Dan Eduard Mihaiescu, Alexandru Mihai Grumezescu, Adina Alberts

**Affiliations:** 1 Department of Science and Engineering of Oxide Materials and Nanomaterials, National University of Science and Technology POLITEHNICA Bucharest, 011061 Bucharest, Romania; antonia.mercan@gmail.com (D.-A.M.); dana.tudorache@upb.ro (D.-I.T.); adelina.niculescu@upb.ro (A.-G.N.); alexandra.birca@upb.ro (A.C.B.); tony.hadibarata@upb.ro (T.H.); grumezescu@yahoo.com (A.M.G.); 2 Research Institute of the University of Bucharest—ICUB, University of Bucharest, 050657 Bucharest, Romania; ariana.hudita@unibuc.ro; 3 Department of Histology, Faculty of Medicine, University of Medicine and Pharmacy of Craiova, 2 Petru Rareş Street, 200349 Craiova, Romania; laurentiu_mogoanta@yahoo.com; 4 Research Center for Microscopic Morphology and Immunology, University of Medicine and Pharmacy of Craiova, 2 Petru Rareş Street, 200349 Craiova, Romania; 5 Department of Pharmacognosy & Phytotherapy, Faculty of Pharmacy, University of Medicine and Pharmacy of Craiova, 2 Petru Rareş Street, 200349 Craiova, Romania; george.mogosanu@umfcv.ro; 6 Drug Research Center, Faculty of Pharmacy, University of Medicine and Pharmacy of Craiova, 2 Petru Rareş Street, 200349 Craiova, Romania; 7 Research Center for Advanced Materials, Products and Processes, National University of Science and Technology POLITEHNICA Bucharest, 060042 Bucharest, Romania; bogdan.vasile@upb.ro; 8 National Research Center for Micro and Nanomaterials, National University of Science and Technology POLITEHNICA Bucharest, 060042 Bucharest, Romania; 9 Department of Biochemistry and Molecular Biology, University of Bucharest, 91-95 Splaiul Independentei Street, 050095 Bucharest, Romania; ionela-cristina.voinea@bio.unibuc.ro (I.C.V.); miruna.stan@bio.unibuc.ro (M.S.S.); 10 Environmental Engineering Program, Faculty of Engineering and Science, Curtin University Malaysia, CDT 250, Miri 98009, Malaysia; 11 Department of Organic Chemistry, National University of Science and Technology POLITEHNICA Bucharest, 011061 Bucharest, Romania; 12 Carol Davila University of Medicine and Pharmacy, 050474 Bucharest, Romania; adina-magdalena.alberts@rez.umfcd.ro

**Keywords:** iron oxide nanoparticles, microfluidic synthesis, double functionalization, antimicrobial activity, silver, copper oxide

## Abstract

This study presents the preparation of hybrid iron oxide nanocomposites through a two-step process combining microfluidic-assisted synthesis and post-synthetic surface modification. Fe_3_O_4_ nanoparticles were synthesized and simultaneously functionalized with salicylic acid using a three-dimensional vortex-type microfluidic chip, enabling rapid and uniform particle formation. The resulting Fe_3_O_4_/SA nanostructures were further modified with either silver or copper oxide to form iron oxide nanocomposites with enhanced antimicrobial functionality. These nanocomposites were subsequently integrated into silica aerogel matrices using a dip-coating approach to improve surface dispersion, structural stability, and biocompatibility. The structural and morphological properties of the samples were investigated using XRD, FT-IR, TEM with SAED analysis, and Raman microscopy. In vitro cytotoxicity and antimicrobial assays demonstrated that Fe_3_O_4_/SA–Ag and Fe_3_O_4_/SA–CuO exhibit potent antibacterial activity and cell type-dependent biocompatibility. In vivo biodistribution studies showed no accumulation in major organs and selective clearance via the spleen, validating the systemic safety of the platform. These findings highlight the potential of the synthesized nanocomposites as biocompatible, antimicrobial coatings for advanced biomedical surfaces.

## 1. Introduction

Over the past decade, the development of nanostructured materials has grown substantially, driven by their potential applications across various technological and biomedical domains [1,2]. Among these, nanoparticles have garnered significant attention due to their tunable physicochemical properties, high surface-area-to-volume ratio, and versatility in functionalization strategies [3,4,5]. The microfluidic technique is an emerging technology that enables the mixing and the manipulation of fluid flows at the microscale, ranging from 10^−^⁹ to 10^−18^ L, whereby physical and chemical processes can be altered as the dimension of the instruments is scaled down to the micrometer level [6,7,8,9]. Compared with traditional methods, microfluidic synthesis is a slightly simpler process to obtain nanoparticles and also has advantages such as excellent fluid control capacity, low sample consumption, and less environmental pollution [6,10,11,12].

Iron oxide nanoparticles, particularly magnetite (Fe_3_O_4_) and maghemite (γ-Fe_2_O_3_), have emerged as prominent candidates in nanomedicine and materials science owing to their biocompatibility, ease of synthesis, and superparamagnetic behavior [3,13]. Due to their permanent magnetization capacity, magnetic nanoparticles (MNPs) are versatile and unique materials and have gained increasing interest, especially γ-Fe_2_O_3_ and Fe_3_O_4_ [14,15,16]. Given their magnetic features, magnetic nanoparticles play an important role in different domains, including contrast agents in magnetic resonance imaging, therapeutic agents for hyperthermia, magnetic fluids, diagnosis and biosensing tools, target and controlled drug delivery systems, data storage applications, inks and magnetic paints, catalysts, microelectronics, magnetic refrigeration tools, batteries, high-density magnetic recording materials, and sorbents for pollutants removal [17,18,19,20,21,22]. Also, specific iron oxide nanoparticle formulations exhibit efficacy in combating microbial infections [23,24].

Despite their promising attributes, Fe_3_O_4_ nanoparticles are inherently prone to oxidation into γ-Fe_2_O_3_ and tend to agglomerate under physiological conditions, limiting their practical utility in biological systems [25]. To combat these drawbacks, it is common to functionalize or cover the particles with a shell on [26,27] made of metals [28], polymers [29,30], or other (organic [31,32] and inorganic [33,34]) stabilizing agents. Such surface modifications can also endow the nanoparticles with additional functionalities, making them suitable for advanced applications, including antimicrobial coatings [35,36].

In this context, our study is focused on engineering hybrid iron oxide-based nanostructures with dual surface functionalization to create multifunctional coatings exhibiting potent antimicrobial activity. Initially, Fe_3_O_4_ nanoparticles were stabilized using salicylic acid—a lipid-soluble organic acid widely used in both human and veterinary medicine—owing to its strong metal-chelating ability and broad-spectrum bioactivity. Salicylic acid has been investigated for its antimicrobial, antibiofilm formation, anti-inflammatory, and antipyretic properties [37,38,39,40].

To enhance their antimicrobial efficacy, the Fe_3_O_4_/SA nanoparticles were further modified with silver and copper oxide nanostructures, due to their well-documented bactericidal and fungicidal properties [41,42]. These hybrid nanocomposites are hypothesized to act via synergistic mechanisms involving reactive oxygen species (ROS) generation, membrane disruption, and metal ion release, enabling their utility in preventing microbial colonization on medical surfaces and devices [43,44].

Silver (Ag) and copper oxide (CuO) nanoparticles have been widely investigated for their broad-spectrum antimicrobial efficacy, attributed to their ability to disrupt microbial membranes, generate ROS, and interfere with intracellular processes. Silver nanoparticles, in particular, exhibit potent bactericidal effects at low concentrations, making them highly effective against both Gram-positive and Gram-negative bacteria [45]. Their mechanism of action involves Ag⁺ ion release, which induces oxidative stress, damages bacterial DNA, and inhibits key enzymes responsible for ATP synthesis and cellular respiration. Furthermore, Ag nanoparticles can penetrate bacterial biofilms—structured microbial communities notoriously resistant to antibiotics—thereby enhancing their therapeutic potential [46].

CuO similarly displays strong antimicrobial properties [41]. CuO nanoparticles exert toxicity through multiple pathways, including membrane destabilization, ROS-mediated lipid peroxidation, and protein oxidation, ultimately resulting in cell death. Additionally, copper ions can interact with nucleic acids and interfere with microbial replication. Notably, both Ag and CuO nanoparticles have demonstrated activity against antibiotic-resistant pathogens such as *Staphylococcus aureus*, *Pseudomonas aeruginosa*, and *Escherichia coli* [47], positioning them as critical tools in the fight against antimicrobial resistance.

This study aims to prepare and characterize antimicrobial coatings based on hybrid iron oxide nanoparticles functionalized with salicylic acid and further modified with silver and copper oxide nanostructures. It also evaluates their structural properties, cytocompatibility, and antimicrobial activity for potential biomedical applications.

## 2. Materials and Methods

### 2.1. Materials

Polymethylmethacrylate (PMMA) sheets, each with a thickness of 2 mm, were utilized in the fabrication of the microfluidic platform, as described in our previous study [17].

The synthesis of nanoparticles was realized by utilizing purchased compounds without any supplementary purification. Iron sulfate heptahydrate, ferric chloride, silver nitrate, copper sulfate pentahydrate, glucose (Sigma Aldrich/Merck, Darmstadt, Germany), salicylic acid (ATOCHIM PROD, Bucharest, Romania), and sodium hydroxide (Lach-Ner, Tovarni, Czech Republic) were utilized in the synthesis process. Ultrapure water was used during all the experimental parts.

### 2.2. Salicylic Acid-Surface Modified Nanoparticles Synthesis

**Figure 1:**
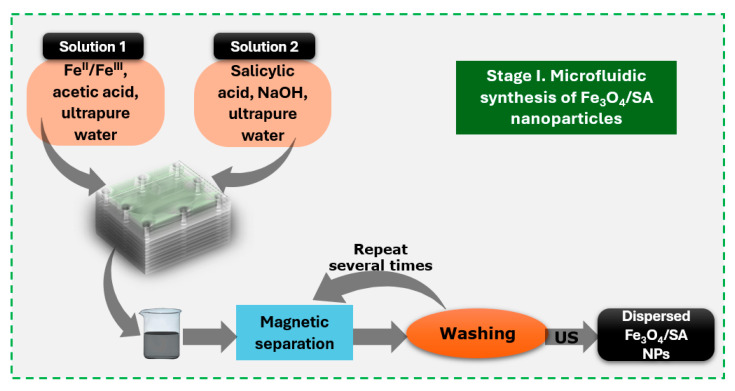
Schematic representation of microfluidic one-shell nanoparticle synthesis.

To obtain functionalized iron oxide nanoparticles (Fe_3_O_4_/SA), two separate precursor solutions were prepared. Solution 1 consisted of FeCl_3_ and FeSO_4_·7H_2_O dissolved in ultrapure water at a molar ratio of Fe^3^⁺:Fe^2^⁺ = 2:1. Solution 2 was prepared by dissolving 2% sodium hydroxide and 1% salicylic acid in 900 mL of ultrapure water. Initially, the microfluidic platform was filled with ultrapure water and degassed to remove any trapped air within the channels. Subsequently, the NaOH/salicylic acid solution (Solution 2) was introduced to condition the system, followed by the injection of the iron precursor solution (Solution 1) using a conventional osmosis pump. This sequential flow enabled controlled mixing and the insitu formation of salicylic acid-functionalized Fe_3_O_4_ nanoparticles within the microchannels. The resulting reaction product was magnetically separated and thoroughly washed with ultrapure water to remove residual reagents. The nanoparticles were then redispersed in ultrapure water via ultrasonication. A schematic overview of the synthesis process is provided in Figure 1 [17].

### 2.3. Hybrid Iron Oxide Nanoparticles

**Figure 2:**
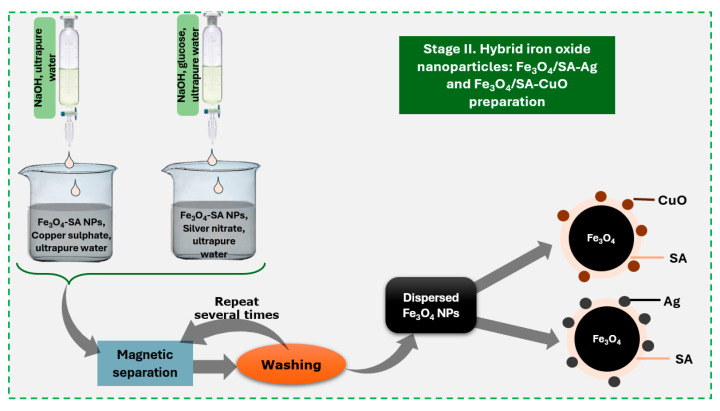
Schematic representation of multiple-shell iron oxide nanoparticle synthesis.

Two types of hybrid iron oxide nanoparticles—Fe_3_O_4_/SA–Ag and Fe_3_O_4_/SA–CuO—were synthesized by introducing silver nitrate and copper sulfate, respectively, into separate aqueous dispersions of preformed Fe_3_O_4_/SA nanoparticles (Figure 2). The amounts of silver and copper precursors were calculated to correspond to a 1:4 mass ratio relative to the iron oxide content, as the secondary surface modification was carried out through a physical adsorption and precipitation process.

For Fe_3_O_4_/SA–Ag, we used a simplified and modified silver nanoparticle synthesis protocol adapted from [48]. A secondary solution was prepared by dissolving 20 g of glucose and 5% (*w*/*v*) sodium hydroxide in ultrapure water, adjusted to a final volume of 120 mL. This reducing solution was continuously and slowly dripped into the silver precursor dispersion under stirring. The reaction mixture reached a final pH of approximately 12.5. The resulting Fe_3_O_4_/SA–Ag nanoparticles were magnetically separated, thoroughly washed with ultrapure water, and redispersed via ultrasonication.

For Fe_3_O_4_/SA–CuO, we used a simplified and modified copper oxide nanoparticle synthesis method adapted from [49]. A third solution consisting of 5% (*w*/*v*) sodium hydroxide in 200 mL of ultrapure water was gradually added to the Fe_3_O_4_/SA–Cu^2^⁺ dispersion under controlled stirring. The pH of the reaction mixture was maintained around 11.5. The resulting Fe_3_O_4_/SA–CuO nanoparticles were magnetically collected, rinsed multiple times with ultrapure water, and dispersed using ultrasonication.

### 2.4. Silica-Based Aerogel Thin Coatings

**Figure 3:**
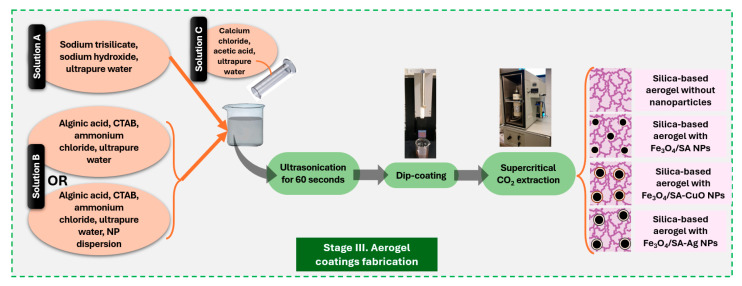
Schematic representation of silica aerogel-based nanostructured thin coatings fabrication.

Coating fabrication was performed following the flowchart outlined in Figure 3. In more detail, two precursor solutions were prepared and combined to obtain a silica-based aerogel matrix without iron oxide nanoparticles. Silica-based aerogel was prepared using a simplified and adapted protocol based on the methods described in [50,51,52].

Solution A was prepared by dissolving 90 g sodium trisilicate and 3 g sodium hydroxide in 400 mL of ultrapure water, yielding a basic silicate solution with a pH of approximately 11.5–12. Solution B consisted of 600 mg alginic acid, 400 mg hexadecyltrimethylammonium bromide (CTAB), and 10 g ammonium chloride, dissolved in 400 mL of ultrapure water, with a pH of approximately 6.5–7.5.

These two solutions were mixed in a 1:1 volumetric ratio (Solution B:Solution A) and homogenized by ultrasonication for 60 s. Separately, a third solution (Solution C) was prepared by dissolving 20 g of calcium chloride and 14 mL of acetic acid in ultrapure water to a final volume of 400 mL, with a pH of approximately 4.5–5. A volume of 5 mL of this calcium-containing solution was then added to the silicate system to initiate crosslinking and gelation.

The final aerogel coatings were fabricated using the dip-coating method onto clean rectangular glass substrates. The coated surfaces were then dried by supercritical CO_2_ extraction to preserve the porous aerogel structure.

For hybrid coatings containing nanoparticles (Fe_3_O_4_/SA, Fe_3_O_4_/SA–Ag, or Fe_3_O_4_/SA–CuO), the only modification to the procedure was the addition of the respective nanoparticle dispersion (200 mg Fe_3_O_4_/SA dispersed in 15 mL ultrapure water) into Solution B before mixing with Solution A. This enabled uniform incorporation of the hybrid nanomaterials into the silica aerogel matrix during coating formation.

### 2.5. Nanoparticle Characterization

#### 2.5.1. X-Ray Diffraction

An X-ray diffraction (XRD) methodology was used to investigate the nanoparticles’ crystal structure, phase composition, and degree of crystallinity. The analysis was conducted using a PANalytical Empyrean model diffractometer (PANalytical, Almelo, The Netherlands), which was equipped with a 2xGe 220 hybrid monochromator on the incident side and a parallel plate collimator coupled to a PIXcel 3D detector on the diffracted side. Grazing incidence X-ray diffraction measurements were carried out at ambient temperature with an incidence angle (ω) of 0.5° and a range of Bragg angles (2θ) from 27° to 80°, utilizing Cu Kα radiation with a wavelength (λ) of 1.5406 Å, under conditions of 40 mA and 45 kV.

#### 2.5.2. Fourier Transform Infrared Spectroscopy

The IR bands were identified via Fourier Transform Infrared (FT-IR) spectroscopy. For this analysis, a Thermo iN10-MX FTIR spectrometer, acquired from Thermo Fisher Scientific (Waltham, MA, USA), was employed. Spectra were collected within the wavenumber range of 4000–400 cm^−1^.

#### 2.5.3. Transmission Electron Microscopy and Selected Area Electron Diffraction

High-resolution transmission electron microscopy (TEM) micrographs were obtained using the Thermo Fisher Scientific 80–200 Titan Themis transmission electron microscope (Hillsboro, OR, USA). The microscope operates at 200 kV in transmission mode, achieving line and point resolutions of 2 Å and 1 Å, respectively. Additionally, crystallographic information was acquired through the selected area electron diffraction (SAED) module, which is integrated into the TEM system.

#### 2.5.4. Cell Viability and Proliferation of Hybrid Iron Oxide Nanoparticles

The cytotoxicity of iron oxide and hybrid iron oxide nanoparticles was tested using two cell lines: renal epithelial (VERO) and human keratinocyte (HaCat) cell lines. The cells were kept in Dulbecco’s Modified Eagle’s Medium (DMEM) for the duration of the experiment, with 1% penicillin-streptomycin mix and 10% fetal bovine serum (FBS) added as supplements. Following enzymatic-chemical trypsin/EDTA treatment to detach the cells from the culture surface, the cells were subsequently seeded into sterile 96-well culture plates at an initial density of 1 × 10^4^ cells/well. After optical microscopy confirmed that the cells had adhered to the culture surface for 24 h, the culture medium was removed and replaced with different concentrations (1 mg/mL, 200 μg/mL, 100 μg/mL, 40 μg/mL, 10 μg/mL, and 1 μg/mL) of the evaluated nanoparticles.

Using an MTT assay, cell viability and proliferation were evaluated using an MTT reagent (i.e., 3-(4,5-dimethylthiazol-2-yl)-2,5-diphenyltetrazolium bromide) (Sigma Aldrich, Merck Group, Darmstadt, Germany). Following a 24 h treatment period, the culture medium was extracted from the cell monolayers and substituted with an MTT solution (1 mg/mL), which was made by dissolving MTT in DMEM without FBS. After the samples had been incubated for four hours at 37 °C, the MTT solution was removed from the cell layers, and the formazan crystals that had formed were dissolved in DMSO. The FlexStation III multimodal plate reader (Molecular Devices, San Jose, CA, USA) was used to measure the optical density of the resultant solution for each sample in triplicate at a wavelength of 550 nm. GraphPad Prism 6 software was used to process the data statistically, utilizing ANOVA (Bonferroni correction). The statistical significance criterion was set at *p* < 0.05, and the data were displayed as the mean of three biological replicates ± the standard deviation.

#### 2.5.5. Antimicrobial Assay

The minimum inhibitory concentration (MIC) was determined using the standard broth microdilution technique in sterile 96-well microtiter plates. The test was conducted in Triptycase Soy Broth (TSB) or simple nutrient broth, with a final volume of 150 µL per well. Serial two-fold (binary) dilutions of each nanoparticle suspension or test compound were prepared directly in the wells. After completing the dilution series, each well was inoculated with 15 µL of bacterial suspension adjusted to a 0.5 McFarland standard (approximately 1.5 × 10^8^ CFU/mL).

The bacterial strains tested were *Staphylococcus aureus* (Gram-positive) and *Escherichia coli* (Gram-negative). To rule out solvent-induced inhibition, solvent controls were prepared in parallel using the same dilution steps applied to the solvent (e.g., DMSO) used to disperse the nanoparticles. Two additional controls were included: a microbial growth control (medium with bacterial suspension but no treatment) and a sterility control (medium only, without bacterial inoculation).

Plates were incubated at 37 °C for 24 h in a humidified chamber. After incubation, bacterial growth was assessed macroscopically and confirmed by spectrophotometric reading at 600 nm. For optical measurement, 100 µL of each sample was transferred into a new sterile 96-well plate before reading. The MIC was defined as the lowest concentration of the tested compound at which no visible bacterial growth or increase in absorbance was observed, compared to the growth control.

### 2.6. Coatings Characterization

#### 2.6.1. Raman Microscopy and Spectroscopy

RAMAN confocal microscopy (Renishaw inVia Raman microscope, Wotton-under-Edge, UK) analysis was performed with a spatial resolution of 0.25–1 µm to obtain information about the distribution of silica-based aerogel coatings. Spectra were acquired using a 532 nm wavelength laser at a laser intensity of 50%. A total of 40 scans were executed for each sample between 200 and 1900 cm^−1^.

#### 2.6.2. In Vitro Cytotoxicity Screening Protocol of Hybrid Iron Oxide Coatings

HaCaT keratinocytes were seeded on UV-sterilized samples represented by coated (SiO_2_-Fe_3_O_4_/SA, SiO_2_-Fe_3_O_4_/SA-Ag and SiO_2_-Fe_3_O_4_/SA-CuO) and uncoated glass slides (which served as controls) at a density of 10^5^ cells/cm^2^ and left to grow for 72 h. 

MTT assay (represented by incubation for 2 h with 1 mg/mL MTT solution and solubilization of formazan crystals with 2-propanol) was used to assess the cell viability at 595 nm (FlexStation, Molecular Device, USA). The inflammation status was investigated by Griess assay (0.1% naphthylethylenediamine dihydrochloride, 1% sulphanilamide in 5% H_3_PO_4_) which measured the level of nitric oxide (NO) in the culture supernatants at 550 nm. In addition, cell membrane damage was evaluated by lactate dehydrogenase (LDH) level kit from Roche Diagnostics (Mannheim, Germany) at 490 nm. Statistical evaluation was performed by one-way ANOVA with Bonferroni post-hoc test (GraphPad Prism 6) on triplicate experiments (*p* < 0.05 was considered significant).

Morphology of actin filaments was observed on Olympus IX71 microscope (Olympus, Tokyo, Japan) after the staining with Alexa Fluor 488-conjugated phalloidin (Invitrogen, Waltham, MA, USA) for 30 min at room temperature the cells previously fixed in 70% ethanol for 15 min and permeabilized with 0.1% Triton X-100.

### 2.7. In Vivo Experimental Model

The experimental protocol was applied according to European Council Directive No. 86/609/24 November 1986, the European Convention on the Protection of Vertebrate Animals (2005), and Romanian Law No. 43/2014 regarding the protection of animals used for scientific purposes. The study was approved by the Ethics Committee of the University of Medicine and Pharmacy of Craiova, Romania (Approval No. 54/19 May 2016).

Three-month-old BALB/c male mice were used for the Fe_3_O_4_/SA sample and for the control. The administration was carried out under general anesthesia. The mice were intraperitoneally inoculated in the groin area with 200 μL of a 1 mg/mL dispersion of Fe_3_O_4_/SA nanostructures obtained in ultrapure water, previously sterilized by UV irradiation for 30 min. Control mice were intraperitoneally inoculated in the groin area with 200 μL of saline.

During the examination period, the mice were housed in the Animal Care Unit of the University of Medicine and Pharmacy of Craiova, maintained in standard conditions (22 ± 2 °C, 55 ± 10% humidity, a 12–hour light–dark cycle, water, and food ad libitum).

Seven days and 14 days after the beginning of the experiment, the animals were euthanized, under general anesthesia, for the sampling of internal organs (brain, myocardium, liver, pancreas, lung, kidney, and spleen). Directly after the sampling, the biological material was washed in phosphate-buffered saline (PBS) to remove blood. Then, the internal organs were fixed in 10% neutral buffered formalin for 72 h at room temperature and processed for the routine histological paraffin embedding technique.

For the histological study, 4 μm-thick serial sections were cut on a MICROM HM355s rotary microtome (MICROM International GmbH, Walldorf, Germany) equipped with a waterfall-based section transfer system (STS, MICROM). The cross sections were placed on histological slides treated with poly-L-Lysine (Sigma-Aldrich, Munich, Germany). After Hematoxylin–Eosin (HE) classical staining, cross sections were evaluated and photographed using a Nikon Eclipse 55i light microscope equipped with a Nikon DS–Fi1 CCD high definition video camera (Nikon Instruments, Apidrag, Romania). Images were captured, stored, and analyzed using Image ProPlus 7 AMS software (Media Cybernetics Inc., Marlow, Buckinghamshire, UK).

## 3. Results

### 3.1. X-Ray Diffraction

**Figure 4:**
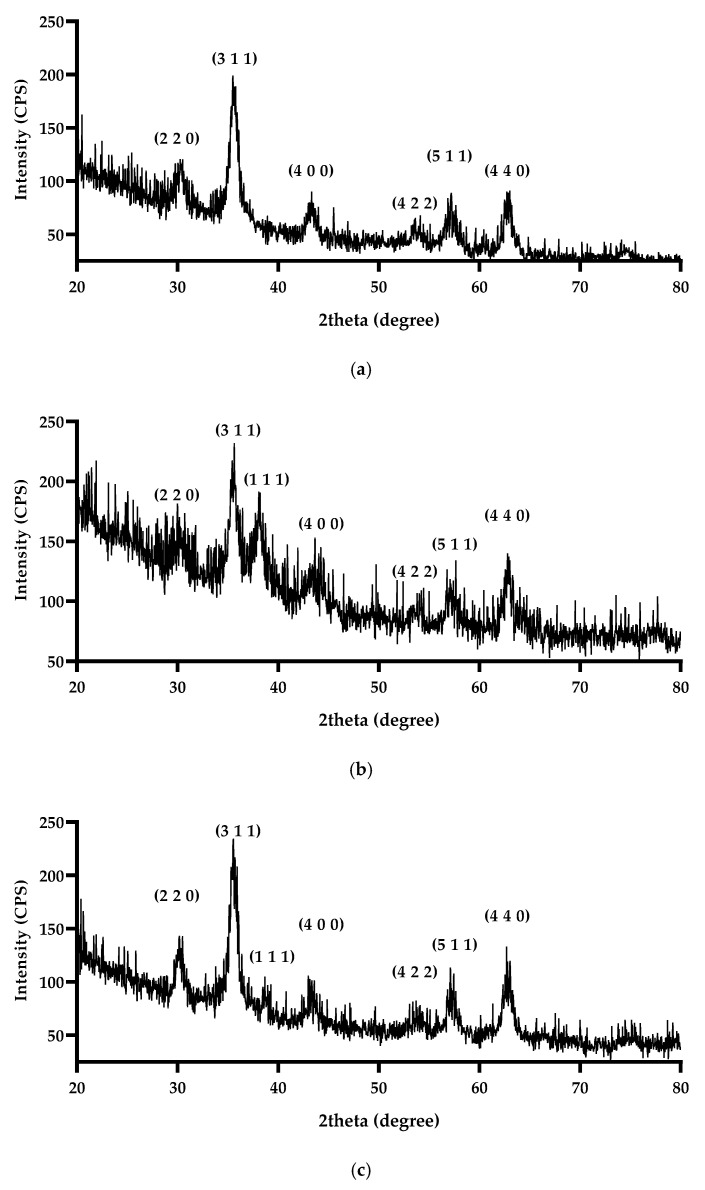
XRD analysis for (**a**) Fe_3_O_4_/SA; (**b**) Fe_3_O_4_/SA-Ag; (**c**) Fe_3_O_4_/SA-CuO powders.

The crystalline structure and phase composition of the synthesized nanomaterials were evaluated using X-ray diffraction (Figure 4). For all three samples—Fe_3_O_4_/SA, Fe_3_O_4_/SA–Ag, and Fe_3_O_4_/SA–CuO—a series of characteristic diffraction peaks were observed at 2θ values corresponding to the (220), (311), (400), (422), (511), and (440) planes. These reflections are in good agreement with the standard pattern for Fe_3_O_4_ with a face-centered cubic inverse spinel structure (JCPDS Card No. 19-0629) [53], confirming the successful formation and preservation of the Fe_3_O_4_ core across all samples. The absence of impurity peaks such as those from γ-Fe_2_O_3_ or hematite indicates the phase purity of the Fe_3_O_4_ core after functionalization.

In the Fe_3_O_4_/SA–Ag sample, an additional diffraction peak was observed at 2θ ≈ 38.1°, which corresponds to the (111) plane of silver nanoparticles, consistent with the face-centered cubic structure (JCPDS Card No. 04-0783) [54].

Similarly, the Fe_3_O_4_/SA–CuO sample exhibited an additional peak around 2θ ≈ 38.7°, which can be attributed to the (111) reflection of monoclinic CuO (JCPDS Card No. 45-0937) [55].

### 3.2. Fourier Transform Infrared Spectroscopy

**Figure 5:**
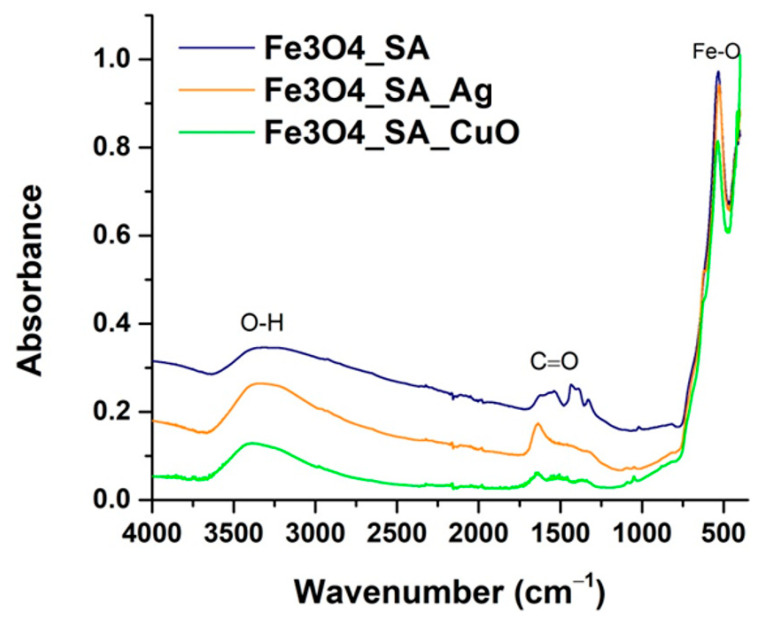
FT-IR spectra of Fe_3_O_4_/SA, Fe_3_O_4_/SA-Ag, Fe_3_O_4_/SA-CuO nanoparticles.

The chemical structure and surface functionalization of the synthesized nanomaterials were investigated by FT-IR spectroscopy, and the spectra of Fe_3_O_4_/SA, Fe_3_O_4_/SA–Ag, and Fe_3_O_4_/SA–CuO are shown in Figure 5.

All three samples exhibit a prominent absorption band in the low-wavenumber region near 550–580 cm^−1^, corresponding to the Fe–O stretching vibration, which is a characteristic fingerprint of the spinel structure of Fe_3_O_4_ nanoparticles. This confirms the presence of the magnetite phase across all hybrid iron oxide samples.

In the Fe_3_O_4_/SA spectrum, broad absorption around 3400 cm^−1^ is attributed to O–H stretching vibrations, indicative of surface-bound hydroxyl groups and/or adsorbed water. Additionally, the clear presence of a band near 1700 cm^−1^ is assigned to C=O stretching from the carboxylic acid group of SA, confirming the successful functionalization of Fe_3_O_4_ with the organic molecule. A weaker band around 2900 cm^−1^, corresponding to C–H stretching vibrations, further supports the incorporation of the aromatic SA moiety on the nanoparticle surface.

Compared to Fe_3_O_4_/SA, both Fe_3_O_4_/SA–Ag and Fe_3_O_4_/SA–CuO display a noticeable decrease in signal intensity within the molecular fingerprint region, particularly around 1700–1300 cm^−1^, where C=O stretching and other aromatic vibrations associated with SA are typically observed. This spectral attenuation is attributed to the presence of Ag and CuO nanostructures on the nanoparticle surface, which likely interfere with the vibrational response of the salicylic acid layer. Residual fingerprint features confirm that salicylic acid remains on the surface, with modified vibrations indicating successful hybridization without complete displacement of the organic layer.

### 3.3. Transmission Electron Microscopy and Selected Area Electron Diffraction 

**Figure 6:**
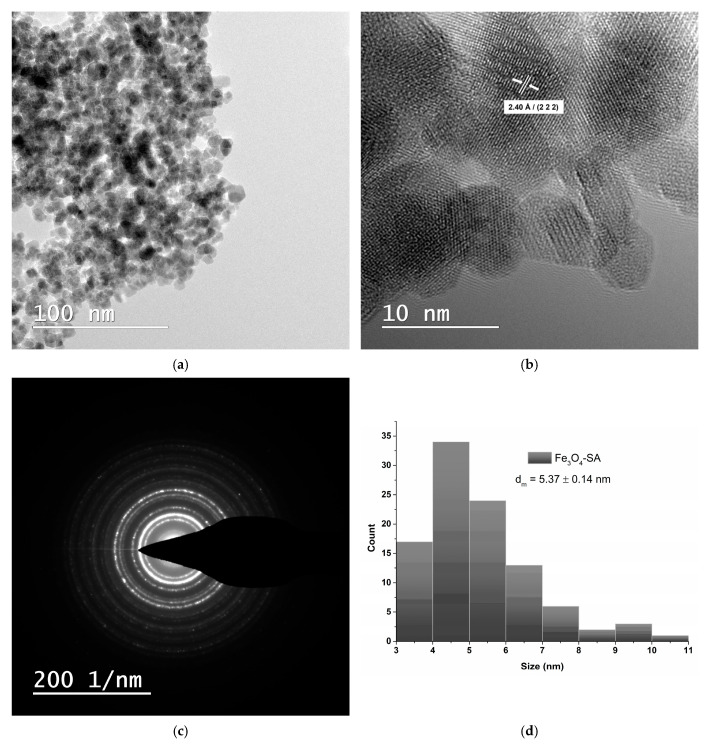
TEM analysis of Fe_3_O_4_/SA nanoparticles: (**a**) brightfield TEM micrograph, (**b**) HR-TEM micrograph, (**c**) SAED analysis, and (**d**) size distribution histogram.

TEM analysis was used to evaluate the morphology, crystallinity, and size of Fe_3_O_4_/SA nanoparticles. The bright field TEM image (Figure 6a) shows that the nanoparticles are roughly spherical, uniformly dispersed, and tend to form loosely packed aggregates, likely due to magnetic interactions. No large-scale agglomeration is observed, indicating good colloidal stability likely conferred by the salicylic acid surface coating. HR-TEM (Figure 6b) reveals the crystalline nature of the particles, with visible lattice fringes. The measured interplanar spacing of 2.40 Å corresponds to the (222) plane of the cubic spinel Fe_3_O_4_ structure, in agreement with XRD results and standard crystallographic data (JCPDS No. 19-0629) [53]. The SAED pattern (Figure 6c) shows concentric diffraction rings, confirming the polycrystalline nature of the Fe_3_O_4_/SA nanoparticles and supporting the well-defined magnetite structure observed in the XRD analysis. The size distribution histogram (Figure 6d), derived from TEM image analysis, shows that most particles fall within the 3–7 nm range, with a calculated mean diameter (d_m_) of 5.37 ± 0.14 nm.

**Figure 7:**
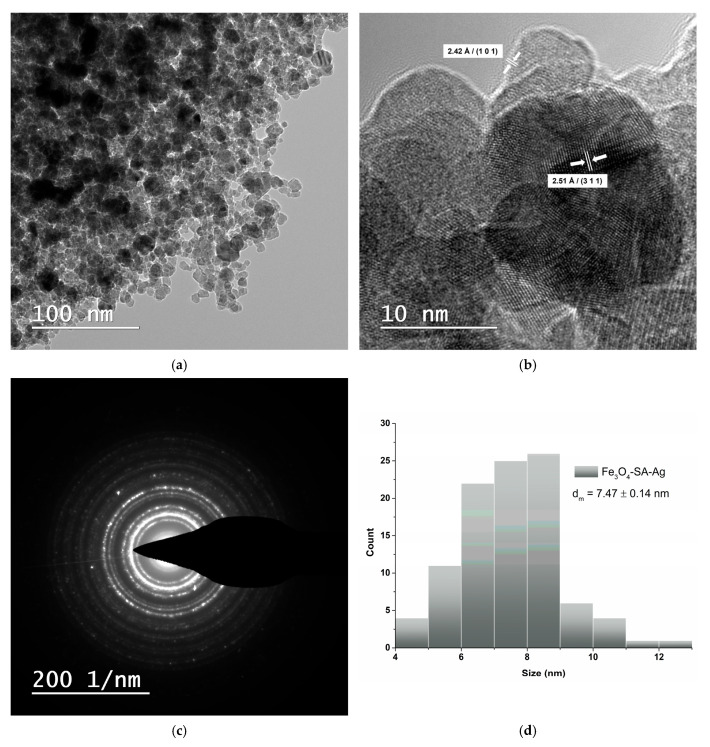
TEM analysis of Fe_3_O_4_/SA-Ag nanoparticles: (**a**) brightfield TEM micrograph, (**b**) HR-TEM micrograph, (**c**) SAED analysis, and (**d**) size distribution histogram.

The TEM images reveal that the Fe_3_O_4_/SA–Ag nanoparticles retain a nearly spherical morphology with moderate aggregation, similar to the Fe_3_O_4_/SA (Figure 7a). High-resolution TEM shows well-defined lattice fringes with interplanar spacings of 2.51 Å and 2.42 Å, corresponding to the (311) plane of Fe_3_O_4_ and the (101) plane of Ag, respectively (Figure 7b). The average particle size increased to 7.47 ± 0.14 nm (Figure 7d), compared to Fe_3_O_4_/SA, reflecting the addition of the Ag. The SAED pattern (Figure 7c) exhibits bright, well-defined rings indicative of a highly crystalline, polycrystalline structure, consistent with the coexistence of both Fe_3_O_4_ and Ag phases and correlating well with the XRD results.

**Figure 8:**
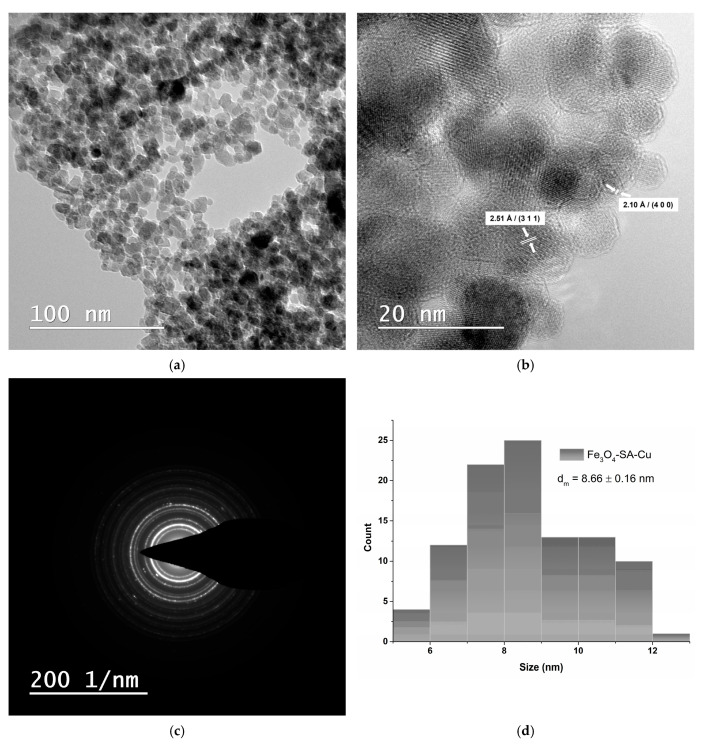
TEM analysis of Fe_3_O_4_/SA-CuO nanoparticles: (**a**) brightfield TEM micrograph, (**b**) HR-TEM micrograph, (**c**) SAED analysis, and (**d**) size distribution histogram.

The Fe_3_O_4_/SA–CuO nanoparticles display a quasi-spherical morphology with moderate clustering (Figure 8a). High-resolution TEM (Figure 8b) reveals well-defined lattice planes with measured interplanar spacings of 2.51 Å and 2.10 Å, corresponding to the (311) plane of Fe_3_O_4_ and the (400) plane of monoclinic CuO, respectively. The average particle size is 8.66 ± 0.16 nm (Figure 8d) while the SAED pattern (Figure 8c) presents well-defined, continuous rings, indicative of a polycrystalline structure, consistent with the co-presence of Fe_3_O_4_ and CuO phases and correlating well with the XRD results.

### 3.4. In Vitro and In Vivo Biocompatibility of Hybrid Iron Oxide Nanoparticles

**Figure 9:**
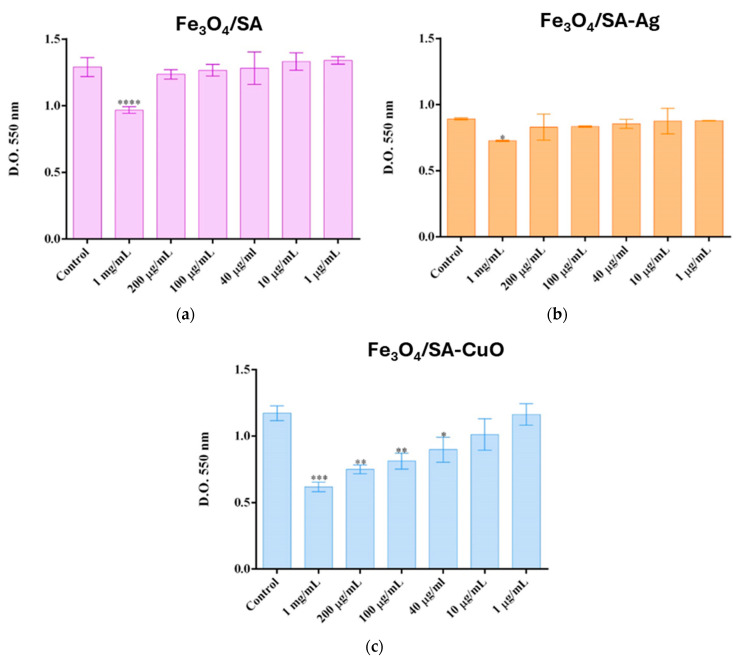
Cell viability of HaCat 24 h after treatment with (**a**) Fe_3_O_4_/SA; (**b**) Fe_3_O_4_/SA-Ag, and (**c**) Fe_3_O_4_/SA-CuO nanoparticles (* *p* ≤ 0.05, ** *p* ≤ 0.01, *** *p* ≤ 0.001, **** *p* ≤ 0.0001 sample vs. control).

**Figure 10:**
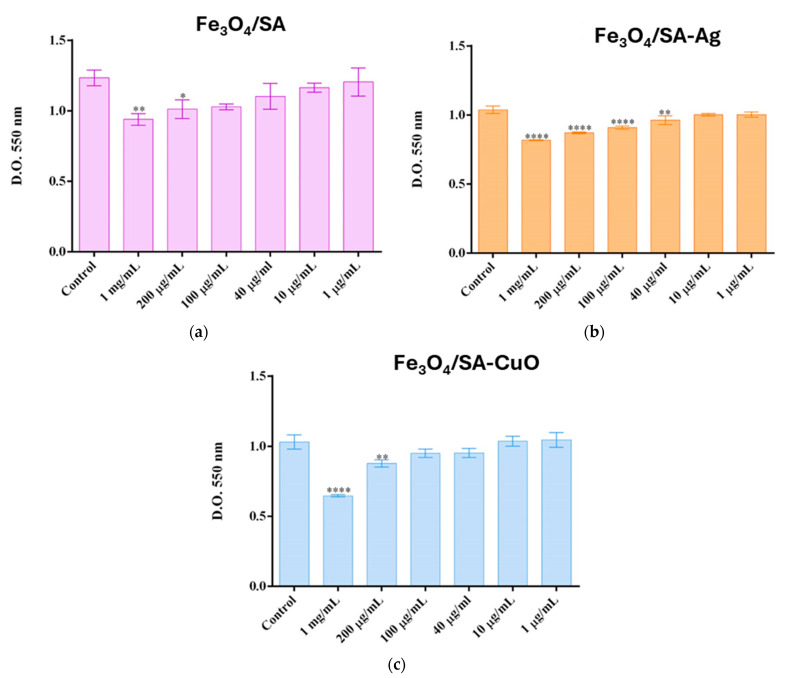
Cell viability of VERO cells 24 h after treatment with (**a**) Fe_3_O_4_/SA; (**b**) Fe_3_O_4_/SA-Ag, and (**c**) Fe_3_O_4_/SA-CuO nanoparticles (* *p* ≤ 0.05, ** *p* ≤ 0.01, **** *p* ≤ 0.0001 sample vs. control).

The cytotoxicity assessment on HaCaT (Figure 9) and VERO (Figure 10) cell lines demonstrated that each nanoparticle type—Fe_3_O_4_/SA, Fe_3_O_4_/SA–Ag, and Fe_3_O_4_/SA–CuO—exhibits a distinct toxicity profile, with biocompatibility dependent on both concentration and cell type.

On the HaCaT line, Fe_3_O_4_/SA nanoparticles were biocompatible at all concentrations ≤ 200 µg/mL, with a significant decrease in viability observed only at 1 mg/mL. Similarly, Fe_3_O_4_/SA–Ag did not induce cytotoxic effects up to 200 µg/mL, suggesting that silver surface modification does not compromise biocompatibility in keratinocytes. In contrast, Fe_3_O_4_/SA–CuO showed higher toxicity, with a statistically significant reduction in viability starting from 40 µg/mL, indicating a more reactive surface and potential oxidative stress response.

In the more sensitive VERO cell line, Fe_3_O_4_/SA reduced viability at concentrations ≥ 200 µg/mL, while Fe_3_O_4_/SA–Ag induced cytotoxicity starting from 40 µg/mL, pointing to a stronger response to silver in renal epithelial cells. Interestingly, Fe_3_O_4_/SA–CuO was better tolerated by VERO cells, with no significant cytotoxicity observed below 200 µg/mL, despite its higher toxicity in HaCaT cells.

These results show that the cytotoxic effect is both dose- and cell type-dependent, and that each nanomaterial presents a different safety threshold. Fe_3_O_4_/SA and Fe_3_O_4_/SA–Ag demonstrate good biocompatibility at concentrations up to 200 µg/mL, particularly for skin-contact applications, while CuO-functionalized systems require careful dose consideration, especially when applied to keratinocyte-rich tissues.

**Figure 11:**
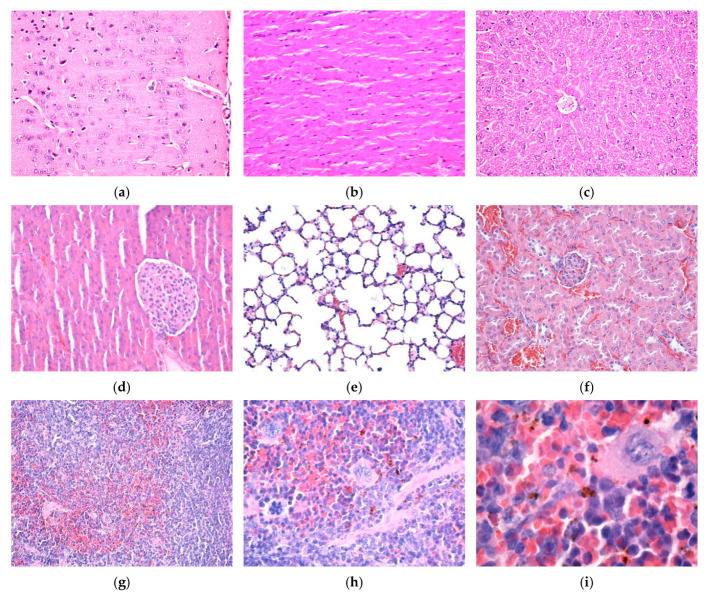
Cross-section through (**a**) brain, (**b**) myocardium, (**c**) liver, (**d**) pancreas, (**e**) lung, (**f**) kidney, and (**g**–**i**) spleen from sample mice injected with Fe_3_O_4_/SA nanostructures and harvested 7 days after intravenous administration. Normal morphology (**a**–**f**). Fe_3_O_4_/SA nanostructures revealed only in the red pulp of the spleen (**g**–**i**). Hematoxylin–Eosin staining: (**a**–**g**) 200×, (**h**) 400×, and (**i**) 1000× magnification.

**Figure 12:**
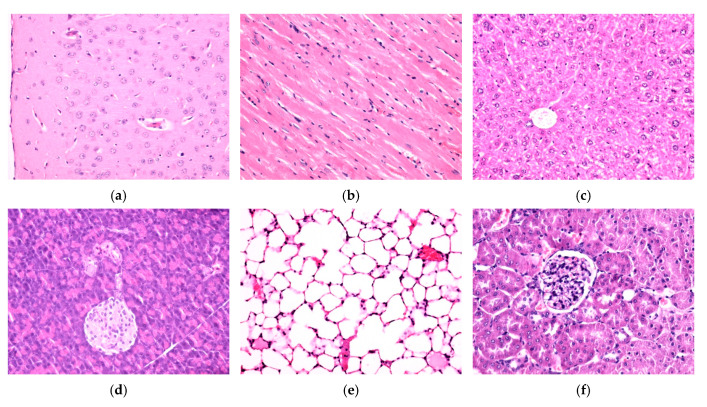

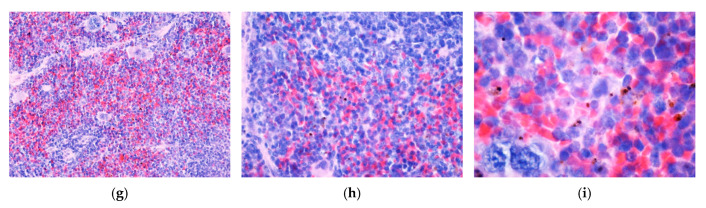
Cross-section through (**a**) brain, (**b**) myocardium, (**c**) liver, (**d**) pancreas, (**e**) lung, (**f**) kidney, and (**g**–**i**) spleen from sample mice injected with Fe_3_O_4_/SA nanostructures and harvested 14 days after intravenous administration. Normal morphology (**a**–**f**). Fe_3_O_4_/SA nanostructures revealed only in the red pulp of the spleen (**g**–**i**). Hematoxylin–Eosin staining: (**a**–**g**) 200×, (**h**) 400×, and (**i**) 1000× magnification.

Fe_3_O_4_/SA nanostructures were not detected in the brain, myocardium, liver, pancreas, lung, and kidney, neither 7 days (Figure 11a–f) nor at 14 days (Figure 12a–f) after intravenous injection. No histopathological alterations of the nervous tissue could be observed, suggesting that NPs do not cross the blood–brain barrier (Figure 11a and Figure 12a).

In the spleen, 7 days and 14 days after intravenous injection, Fe_3_O_4_/SA nanostructures were only evident in the red pulp. At 14 days, NPs were detected in higher concentration than in the sample collected at 7 days. In the white pulp, NPs were absent. However, hypertrophy of the white pulp was observed because nanoparticles stimulated the formation of macrophages with multi-lobular nuclei. In the red pulp, NPs were evident in the cells of the macrophage system, both in the Billroth cords and in the sinusoidal capillaries. The NPs appeared as granular, agglomerated, spherical structures of variable size, with a diameter of up to 3 μm, of brownish-blackish color. The density of the NPs varied from one cell to another, some cells presenting a greater amount of endocytosed NPs (Figure 11g–i and Figure 12g–i).

### 3.5. Antibacterial Characterization

**Figure 13:**
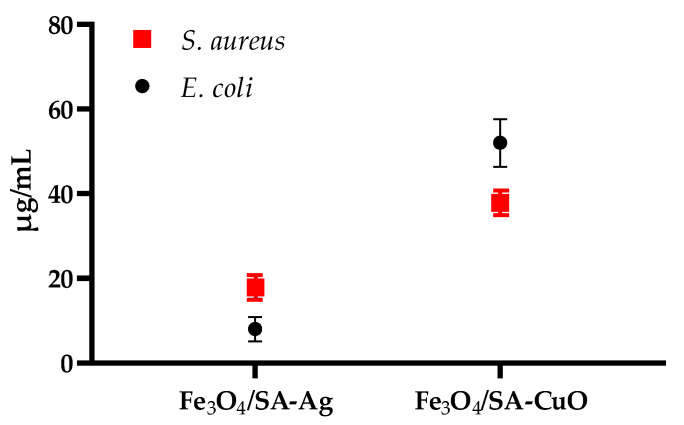
MIC values of Fe_3_O_4_/SA–Ag and Fe_3_O_4_/SA–CuO nanocomposites against *S. aureus* and *E. coli*.

The antimicrobial activity of Fe_3_O_4_-based nanostructures was evaluated against *Staphylococcus aureus* and *Escherichia coli* by determining their minimum inhibitory concentrations, as shown in Figure 13. All nanocomposites displayed dose-dependent antibacterial effects, with significant variation based on both the type of surface functionalization and the bacterial strain.

For *S. aureus*, Fe_3_O_4_/SA exhibited modest antimicrobial activity, with an MIC of approximately 250 µg/mL, likely due to mild ROS generation by Fe_3_O_4_ and the inherent antibacterial properties of salicylic acid. Functionalization with Ag or CuO significantly enhanced efficacy, with Fe_3_O_4_/SA–Ag and Fe_3_O_4_/SA–CuO demonstrating MIC values of approximately 18 µg/mL and 38 µg/mL, respectively.

In the case of *E. coli*, Fe_3_O_4_/SA showed minimal inhibitory effect, with a much higher MIC of approximately 500 µg/mL, underscoring its limited action on Gram-negative bacteria. By contrast, Fe_3_O_4_/SA–Ag exhibited the strongest antibacterial effect, with an MIC of only 8 µg/mL, while Fe_3_O_4_/SA–CuO required a higher MIC of about 52 µg/mL to inhibit growth. These findings suggest that silver-functionalized systems are particularly effective against Gram-negative pathogens, likely due to their ability to penetrate the outer membrane and disrupt intracellular targets.

Overall, Fe_3_O_4_/SA–Ag demonstrates the most potent and broad-spectrum antimicrobial profile, while Fe_3_O_4_/SA–CuO shows preferential activity toward *S. aureus*, reflecting known differences in bacterial wall composition and susceptibility.

### 3.6. Silica-Aerogel-Based Coatings Characterization

**Figure 14:**
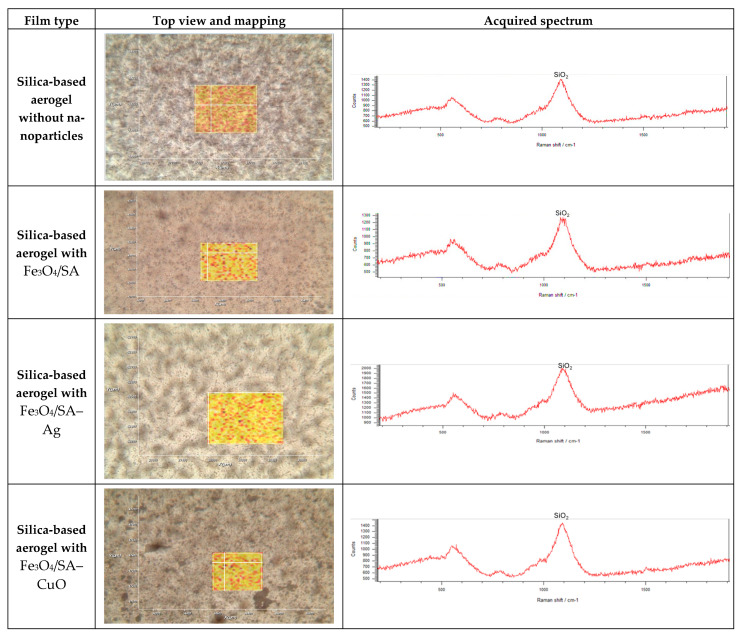
Raman analysis results for the evaluated aerogel-based thin coatings.

Raman spectroscopy and mapping were used to evaluate the structural features and homogeneity of the silica-based coatings. All four samples—SiO_2_, SiO_2_/Fe_3_O_4_/SA, SiO_2_/Fe_3_O_4_/SA–Ag, and SiO_2_/Fe_3_O_4_/SA–CuO—displayed similar Raman spectral profiles (Figure 14).

The Raman spectra are characterized by a strong and broad peak centered around ~1100 cm^−1^, which is attributed to the Si–O–Si asymmetric stretching vibration, confirming the presence of amorphous silica (SiO_2_) as the dominant matrix. A secondary, broader feature near ~550 cm^−1^ corresponds to Si–O bending modes, further validating the silica network. No significant shifts or new peaks were observed after embedding Fe_3_O_4_, Ag, or CuO components, suggesting that the incorporation of nanoparticles does not disrupt the silica framework at the molecular level.

The corresponding Raman image maps confirm the homogeneous distribution of the silica matrix across the analyzed surface area. The uniform Raman signal across the scanned area confirms consistent material dispersion and coating, indicating that the silica aerogel structure remains intact and continuous after nanoparticle incorporation.

### 3.7. In Vitro Behaviour of Human Keratinocytes on Thin Coatings

**Figure 15:**
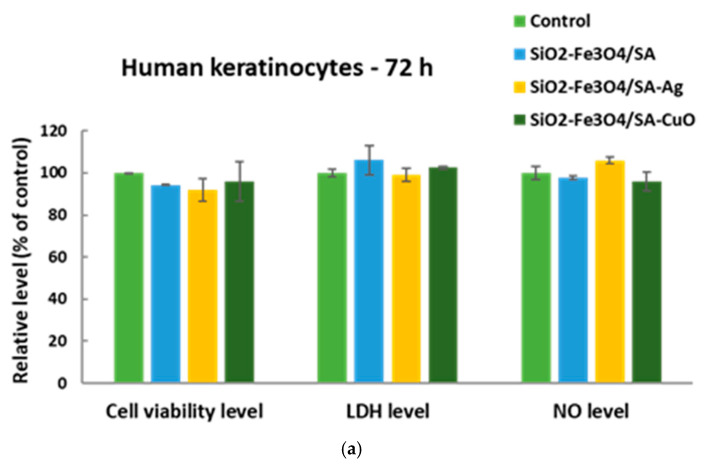

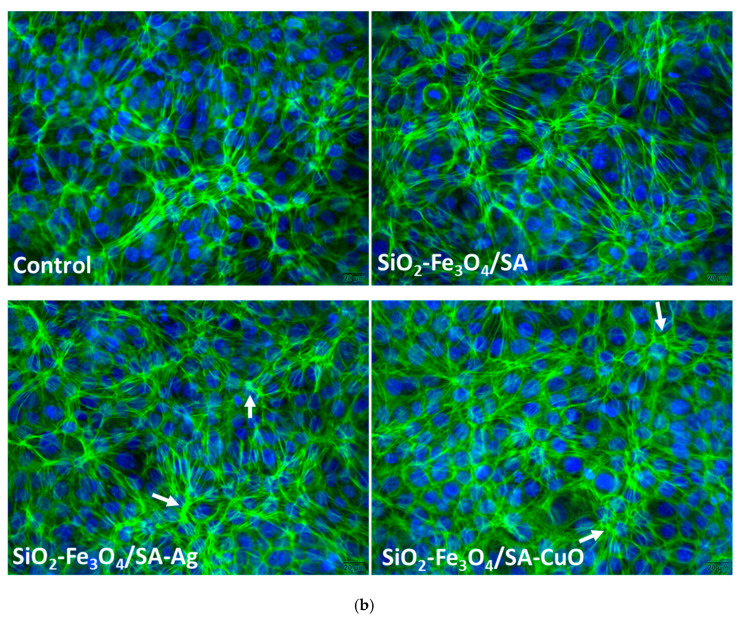
(**a**) In vitro behavior of human keratinocytes after 72 h of cell growth on the surfaces of SiO_2_-Fe_3_O_4_/SA, SiO_2_-Fe_3_O_4_/SA-Ag, and SiO_2_-Fe_3_O_4_/SA-CuO, measured by cell viability, LDH release, and NO production; and (**b**) phase-contrast images. Results are represented relative to control (uncoated glass slides), being means ± standard deviation (n = 3). Cell adherence to these coatings was revealed by F-actin staining (green) with phalloidin-FITC dye (nuclei counterstained in blue with DAPI). Note the adhesion zippers indicated by white arrows. Images were captured with 40× objective. Scale bar is 20 µm for all images.

In vitro measurements on keratinocytes (Figure 15a) revealed cell viability levels higher than 90% of the control for all tested coatings, demonstrating their good biocompatibility. The 8% decrease in the case of SiO_2_-Fe_3_O_4_/SA-Ag could be correlated with the 6% increase in NO production after 72 h, suggesting a slight inflammatory potential, most probably due to silver ions, as previously described for Ag nanoparticles on human primary keratinocytes [56]. However, no cell membrane damage was recorded for both SiO_2_-Fe_3_O_4_/SA-Ag and SiO_2_-Fe_3_O_4_/SA-CuO samples, the levels being near the control ones. In addition, these results are in accordance with previous data reporting low toxicity of CuO-based nanostructures on human keratinocytes [57].

Furthermore, the exploration of F-actin organization in human keratinocytes (Figure 15b) showed the formation of a robust network of actin stress fibers along with the presence of adhesion zippers (arrows), which serve as intermediates in the maturation of adherens junctions, bringing adjacent membranes together and maintaining epithelial integrity. Actin filaments were concentrated and formed a honeycomb of cortical actin belts, which are essential in achieving stable cell contacts. The formation of radial actin fibers is an indicator of a good model for cell stratification, dependent upon calcium-induced intercellular contacts [58]. This was probably supported in our study by the biocompatible coatings of SiO_2_-Fe_3_O_4_/SA-Ag and SiO_2_-Fe_3_O_4_/SA-CuO.

Taken together, our results suggest that the tested samples support human keratinocytes’ viability and adhesion, offering promising applications for skin regeneration and wound healing.

## 4. Discussion

The present study follows our previous work [17], which successfully demonstrated the rapid, microfluidic-assisted synthesis of iron oxide nanoparticles functionalized with salicylic acid. Compared to traditional co-precipitation methods, the microfluidic platform ensures superior control over particle size, monodispersity, and reaction kinetics, allowing for reproducible synthesis of uniform nanoparticles suitable for biomedical applications [59,60,61]. In the current study, we advanced this platform by first synthesizing Fe_3_O_4_ nanoparticles via microfluidics and functionalizing them in situ with salicylic acid, followed by distinct post-synthetic surface decoration with either Ag or CuO nanostructures. Through this multi-step process, we obtained two hybrid nanomaterials—Fe_3_O_4_/SA-Ag and Fe_3_O_4_/SA-CuO—designed to enhance antimicrobial efficacy while improving biocompatibility compared to bulk Ag and CuO. While Ag and CuO nanoparticles are widely recognized for their strong antimicrobial activity [62,63], their cytotoxicity remains a major concern [64,65,66,67]. AgNPs can induce mitochondrial dysfunction, ROS overproduction, and DNA fragmentation in mammalian cells at doses as low as 10–25 µg/mL [68,69]. CuO nanoparticles, though less commonly used, have been shown to cause even more pronounced toxicity through Fenton-like reactions, triggering intracellular oxidative stress and membrane lipid peroxidation [70,71]. These dose-dependent effects limit their standalone use in biomedical coatings.

To overcome the limitations associated with the cytotoxicity of Ag and CuO nanoparticles, we prepared hybrid Fe_3_O_4_-based nanoparticles by functionalizing the iron oxide core with salicylic acid, followed by decoration with Ag and CuO. This configuration minimizes metal ion leaching and reduces direct nanoparticle–cell interaction from the Ag and CuO components, thus mitigating their cytotoxicity. Our cytotoxicity results confirmed that Fe_3_O_4_/SA–Ag and Fe_3_O_4_/SA–CuO nanocomposites exhibited improved biocompatibility compared to bulk Ag and CuO nanoparticles, with no significant reduction in HaCaT cell viability at concentrations up to 200 µg/mL, and variable tolerance in VERO cells, depending on the formulation. These findings are consistent with previous reports on hybrid systems designed to reduce ion release and nanoparticle–cell contact [72,73]. The cytotoxicity profile was cell type-dependent: HaCaT keratinocytes showed higher tolerance to Fe_3_O_4_/SA–Ag, while VERO cells were more sensitive, particularly to Ag-functionalized coatings. This highlights the importance of cell-specific responses when evaluating nanoparticle safety and underscores the need for application-specific material optimization.

Additionally, in vivo biodistribution studies confirmed the absence of Fe_3_O_4_/SA accumulation in vital organs such as the brain, liver, and kidney, supporting the systemic safety of these nanostructures. Their selective localization in the spleen, particularly within macrophages in the red pulp, suggests clearance through the reticuloendothelial system (RES) without triggering tissue damage. This distribution pattern aligns with previous studies reporting that iron oxide nanoparticles tend to accumulate in the spleen and liver following intravenous injection, depending on their size, coating, and surface charge [74,75,76].

Importantly, the lack of brain accumulation observed in our study further supports the notion that Fe_3_O_4_/SA nanoparticles do not cross the blood–brain barrier, which is consistent with findings from [77,78] and confirms their suitability for systemic applications where central nervous system exposure is undesirable. Compared to some earlier reports where uncoated or unstable iron oxide nanoparticles induced hepatic or renal retention and oxidative stress, our results indicate that salicylic acid surface functionalization may improve biodistribution profiles and minimize off-target organ accumulation.

From an antimicrobial perspective, the hybrid nanomaterials displayed differential but effective antibacterial activity against both *Staphylococcus aureus* and *Escherichia coli*, with Fe_3_O_4_/SA-CuO exhibiting the strongest inhibition, followed by Fe_3_O_4_/SA-Ag, while Fe_3_O_4_/SA alone showed only mild activity, primarily against *S. aureus*. The limited activity of Fe_3_O_4_/SA may be attributed to modest ROS generation by Fe_3_O_4_ and the intrinsic antimicrobial properties of salicylic acid, which can interfere with bacterial membrane integrity and biofilm formation.

The enhanced efficacy of the Ag- and CuO-functionalized systems can be attributed to the distinct antimicrobial mechanisms of Ag⁺ and Cu^2^⁺ ions. In *S. aureus*, both ions interfere with peptidoglycan synthesis, membrane stability, and inhibit thiol-dependent enzymes involved in energy metabolism [47,79]. In *E. coli*, their small size and membrane-penetrating ability allow them to breach the outer membrane, resulting in intracellular ROS production, protein oxidation, and DNA damage [45,80].

To translate the bioactivity and stability of the Fe_3_O_4_-based nanocomposites into practical biomedical coatings, we further integrated the system into a silica aerogel matrix—a platform known for its versatility in biomedical applications. The incorporation of a silica aerogel network provided several advantages. Silica aerogels are valued for their ultrahigh porosity, biocompatibility, and tunable surface chemistry, which support both cell compatibility and drug-loading capacity [81,82]. In our system, the aerogel served as a stabilizing scaffold, preventing nanoparticle agglomeration and ensuring uniform distribution across the coating surface. This homogeneous dispersion enhances antimicrobial surface contact while also contributing to mechanical durability, chemical stability, and reduced cytotoxicity [83,84]. Additionally, the inert and non-immunogenic nature of the silica matrix may help minimize inflammatory responses, supporting its application in sensitive environments such as wound dressings, implant surfaces, or diagnostic interfaces [85,86]. The modularity of the aerogel system also enables future integration of additional therapeutic agents, growth factors, or responsive layers, allowing for multifunctional and customizable surface designs tailored for advanced biomedical use [87,88].

## 5. Conclusions

In this study, we successfully synthesized Fe_3_O_4_ nanoparticles functionalized with salicylic acid using a microfluidic platform, followed by post-synthetic surface modification with either Ag or CuO to obtain hybrid nanosystems. This two-step approach led to the development of hybrid nanosystems—Fe_3_O_4_/SA–Ag and Fe_3_O_4_/SA–CuO—designed to enhance antimicrobial activity while reducing the cytotoxic effects commonly associated with bulk Ag and CuO nanoparticles. In vitro cytotoxicity assays demonstrated a cell type-dependent response: Fe_3_O_4_/SA–Ag was well tolerated by HaCaT keratinocytes at concentrations up to 200 µg/mL, while Fe_3_O_4_/SA–CuO induced greater sensitivity in this cell line but showed improved compatibility in VERO epithelial cells. Antibacterial testing revealed that Fe_3_O_4_/SA–CuO displayed the most potent activity against both *S. aureus* and *E. coli*, consistent with its higher potential for ROS generation and membrane disruption. In vivo studies showed no accumulation of Fe_3_O_4_/SA nanostructures in major organs and no associated tissue damage, with selective localization in the spleen indicating safe clearance via the reticuloendothelial system. The silica aerogel matrix enhanced nanoparticle stability, dispersion, and biocompatibility, supporting the development of effective antimicrobial coatings. These results highlight the potential of Fe_3_O_4_-based hybrids in biomedical applications, with future work focused on wound healing models and multifunctional therapeutic integration.

## Data Availability

Data are contained within the article.
